# Intra-Articular Osteoid Osteoma: Radiological Manifestations

**DOI:** 10.5334/jbsr.2040

**Published:** 2020-05-06

**Authors:** Najoi Chatt, Pierre-Louis Docquier, Dana Dumitriu

**Affiliations:** 1 Clinique universitaire St Luc, BE

**Keywords:** intra-articular osteoid osteoma, synovitis, arthritis, imaging

## Abstract

We report a case of intra-articular osteoid osteoma (IAOO) of the elbow, in order to point out the clinical and imaging features which made the case challenging and caused a diagnostic delay.

## Case Report

**Figure 1:**
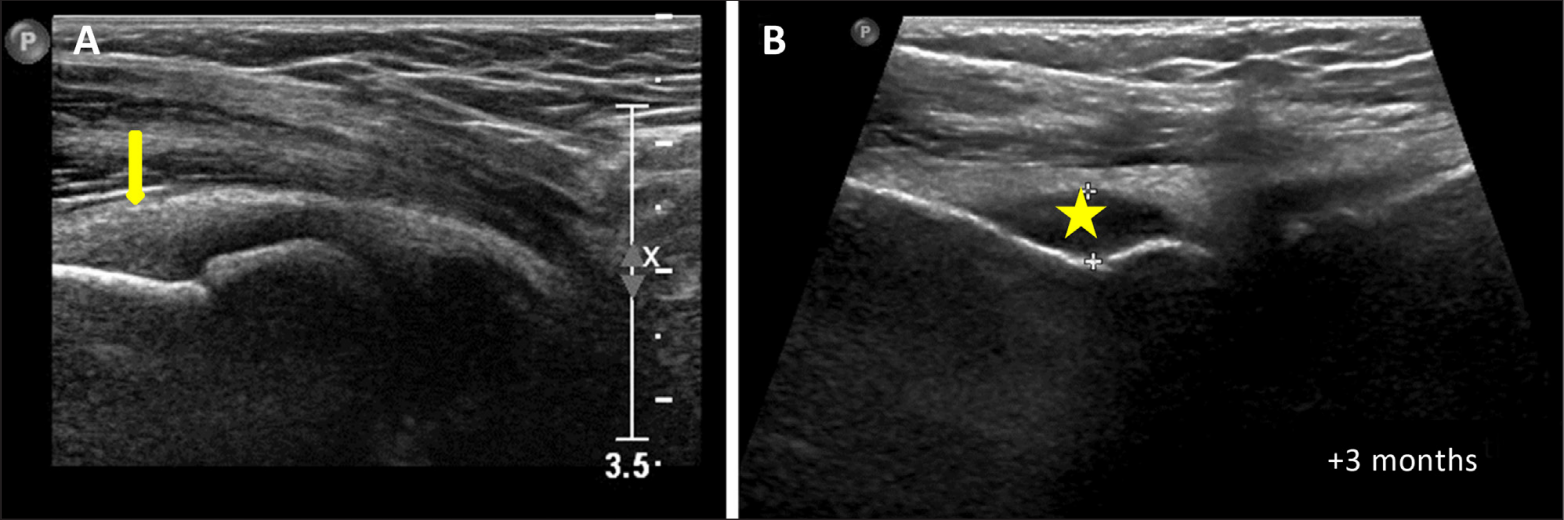


A 9-year-old boy presented to the rheumatology consultation with left elbow pain for the prior three months. The pain was associated with swelling, stiffness, and limitation of movement suggestive of inflammation. The blood work was normal. Mono-articular juvenile idiopathic arthritis (JIA) was suspected, and the patient was referred for left elbow ultrasound, which confirmed synovial thickening (arrow on Figure 1A). Treatment with nonsteroidal anti-inflammatory drugs was started, and three weeks later an intra-articular infiltration with cortico-steroids was carried out.

**Figure 2:**
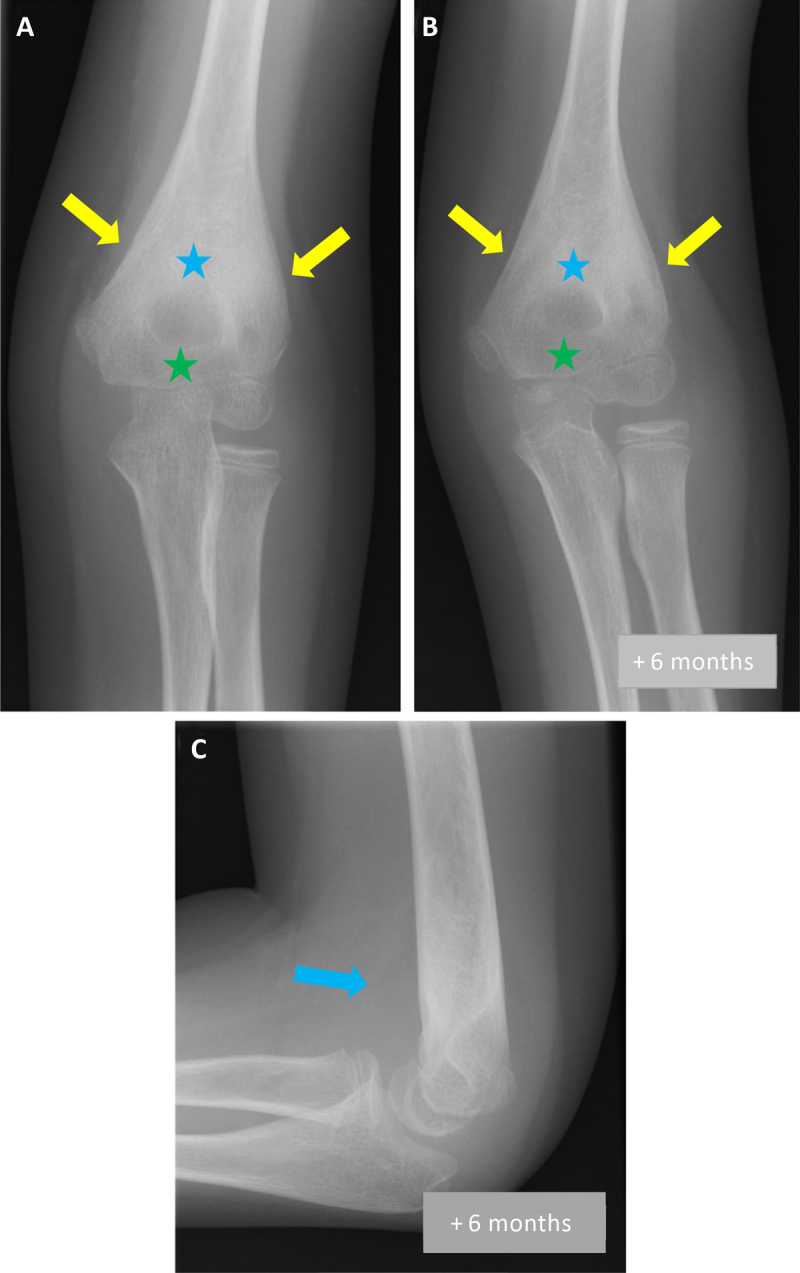


Three months later, as the symptoms persisted, a new ultrasound of the elbow was performed, demonstrating joint effusion (asterisk on Figure 1B); elbow radiograph on the same day showed metaphyseal sclerosis (blue asterisk on Figure 2A), peri-articular osteopenia (green asterisk on Figure 2A) and periosteal apposition (arrows on Figure 2A). Septic arthritis was suspected and a joint lavage was performed; bacteriological samples were negative. Treatment with ledertrexate for chronic synovitis was started.

Six months later, the swelling and the limitation of movement persisted. X-ray showed an increase in metaphyseal sclerosis (blue asterisk on Figure 2B) and peri-articular osteopenia (green asterisk on Figure 2B), incorporation of periosteal apposition (arrows on Figure 2B) and intra-articular fluid (arrow on Figure 2C). Given these findings, the diagnosis of JIA was reconsidered. Repeat blood tests revealed an inflammation with slightly increased C-reactive protein (CRP) and leukocytosis. These results, in correlation to the X-ray, raised the suspicion of chronic osteomyelitis and magnetic resonance imaging (MRI) of the elbow was performed.

**Figure 3:**
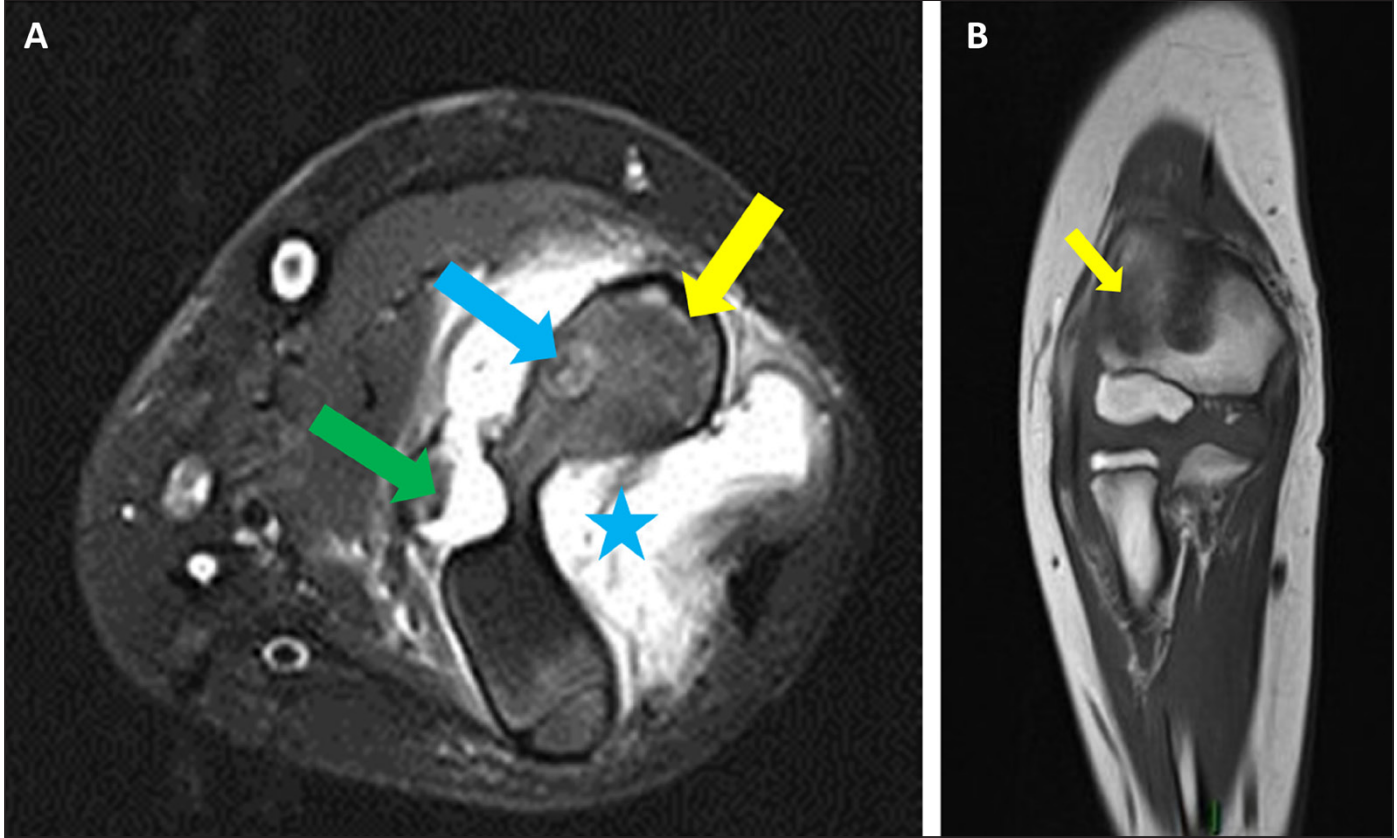


MRI demonstrated joint effusion (blue asterisk on Figure 3A), bone marrow edema in the distal humerus (yellow arrows on Figure 3A, B) and a lesion in the lateral metaphysis with heterogenous signal intensity on T2-weighted images with fat saturation, corresponding to a possible abscess or nidus (blue arrow, Figure 3A). Soft tissues around the joint were infiltrated (green arrow, Figure 3A). These findings were considered suggestive of chronic osteomyelitis or osteoid osteoma. Synovectomy and surgical resection of the focal lesion were performed. Pathological examination finally settled the diagnosis of intra-articular osteoid osteoma (IAOO).

## Comment

Osteoid osteoma is a benign bone tumor, commonly occurring in the diaphysis of long bones, with a characteristic clinical and radiological presentation.

IAOO is less common (10–13% of osteoid osteomas), involving commonly the hip and very rarely the elbow. Because of the intra-articular location of the tumor, stiffness, pain and decreased mobility are the most common symptoms, simulating arthritis.

Whereas the classical radiological presentation of osteoid osteoma is that of a rich osteosclerotic process along the diaphysis of a long bone and centered on a small osteolytic nidus, the imaging features of IAOO are often atypical and lead to a delay in diagnosis. In IAOO, the osteosclerotic reaction is less abundant, sometimes absent. Osteopenia may appear, because of the articular inflammation associated with IAOO. The nidus is more difficult to detect because of its osteopenic surroundings.

Even though it was not performed in our case, CT is the best modality to identify the nidus in osteoid osteoma, once the suspected area of interest has been identified. CT is the method of choice to characterize bone changes and may differentiate between osteoid osteoma and chronic osteomyelitis. The role of MRI is controversial because of the variable appearance of the lesion and the reactive changes in the bone marrow and soft tissues [1].

This case shows the importance of identifying atypical X-ray manifestations in the context of arthritis. The lack of response to adequate treatment must suggest an alternative diagnosis and the search for a possible osteoid osteoma in pediatric patients.

## References

[1] Cotta AC, Teles de Melo R, et al. Diagnostic difficulties in osteoid osteoma of the elbow: Clinical, radiological and histopathological study. Radiol Bras. 2012; 45(1): 13–19. DOI: 10.1590/S0100-39842012000100005

